# Supplementary material for: Dopamine improves defective cortical and muscular connectivity during bilateral control of gait in Parkinson’s disease
Source: Commun Biol. 2024 Apr 24;7:495. doi: 10.1038/s42003-024-06195-5 (PMC11043351; doi:10.1038/s42003-024-06195-5)
Supplement: Supplementary file 2 — Supplementary information [file 42003_2024_6195_MOESM2_ESM.pdf]

Dopamine improves defective cortical and muscular connectivity during bilateral control of gait in Parkinson's Disease

Paulo Cezar Rocha dos Santos[1,2,3,#]; Benedetta Heimler[2]; Or Koren[2]; Tamar Flash[1]; Meir Plotnik[2,4,5]*

[1]Department of Computer Science and Applied Mathematics, Weizmann Institute of Science, Rehovot, Israel

[2]Center of Advanced Technologies in Rehabilitation, Sheba Medical Center, Ramat Gan, Israel

[3]IDOR/Pioneer Science Initiative, Rio de Janeiro, Rio de Janeiro, Brazil

[4]Sagol School of Neuroscience, Tel Aviv University, Tel Aviv, Israel

[5]Department of Physiology and Pharmacology, Sackler Faculty of Medicine, Tel Aviv University, Tel Aviv, Israel

[#] **1st Corresponding author:** Department of Computer Science and Applied Mathematics, Weizmann Institute of Science, Rehovot, Israel

E-mail: paulo-cezar.rocha-dos-santos@weimann.ac.il; paulcezarr@hotmail.com

*** 2nd Corresponding author:** Center of Advanced Technologies in Rehabilitation, Sheba Medical Center, Ramat Gan, Israel.

E-mail: meir.plotnik@sheba.health.gov.il

Supplementary Information Contents

Supplementary Text 3
Supplementary Note 1 - Gait harmony 3
Supplementary Note 2 - Stepping Phase Coordination Index (PCI) 4
Supplementary Note 3 - Statistics 5
Correlation 5
Supplementary Results 6
Supplementary Tables 7
Supplementary Table 1. 7
Supplementary Table 2. 8
Supplementary Figures 9
Supplementary Figure 1. 9
Supplementary Figure 2. 9
Supplementary Figure 3. 10
Supplementary Figure 4. 10
Supplementary Figure 5. 11
Supplementary Figure 6. 12
Supplementary Figure 7. 13
Supplementary Figure 8. 14
Supplementary Figure 9. 15
Supplementary Figure 10. 16
Supplementary References 16

Supplementary Text

This material includes additional information on the study entitled “Dopamine improves defective cortical and muscular connectivity during bilateral control of gait in Parkinson's Disease”. In this study, we aimed: 1) to compare cortico-cortical, cortico-muscular, and intermuscular couplings and gait outcomes (gait scores and harmony (measured as the golden ratio – detailed below), step-time coordination) between the More Affected Side (MAS) and Less Affected Side (LAS) in people with Parkinson’s Disease (PD) in ON (PD-ON) and OFF (PD-OFF) medication states vs. healthy control older adults (OA). 2) We correlate medication effect on bilateral coherence with gait outcomes and Parkinson’s Disease (PD) symptoms when Medication (PD-ON and PD-OFF) and Side (MAS and LAS) were significantly different.

In this supplementary material, we included details in the secondary analysis (Gait harmony and Stepping Phase Coordination Index - PCI), Statistics, and Results in addition to the main manuscript.

Supplementary Note 1 - Gait harmony

The capacity of humans to transfer the symmetry during cycled movements, such as gait, in synchronized, symmetric, and rhythmic movements warrants gait harmony[1,2]. One way to estimate gait harmony is by computing the proportion between even and odd harmonics or via the proportion between two segments (e.g., the swing and stance phases) recorded within the same gait cycle[1,3,4]. Evidence has consistently shown that, during comfortable speed human gait, the proportion of stance and swing phase varies from 60–62% versus 40–38% of the gait cycle. The maintenance of such harmony between swing and stance segments is a physiological marker of comfortable gait in healthy younger individuals. Opposingly, this proportion is not maintained in pathological gait (e.g., Parkinson’s Disease, Cerebellar ataxia, and cerebral palsy)[1,3,4].

Curiously, the proportion ratio between the stance and swing phase durations is close to the golden ratio (ϕ, an irrational number –1.618034). The golden ratio was reported in III century BC by Euclid, which involved dividing a given straight line in such a way that the ratio between the shorter and the longer segments is equal to the ratio between the longer segment and the entire line. A seemingly disparate variety of physical and biological systems (e.g., plant stems and seed arrangement on flower heads, spiral structures of galaxies and mollusks,

quantum phase transitions, etc[5–7]) have displayed similar harmonic characteristics related to ϕ. This harmonic proportion was previously observed during "health" gait but not during pathological gait, such as in PD[1,2]. It is thus reasonable to assume that the loss of harmony may be linked to neurological impairments of PD. We thus investigated whether the cortico-muscular control of gait may reflect impairments in the maintenance of gait harmony, as well as in step timing gait coordination (measured by Phase Coordination Index) in PD.

In our study (also indicated in the main document). The ϕ was computed by the following formula, which calculates the ratio between the swing (i.e., shorter segment) and stance (i.e., longer segment) phase durations, assuming that this result should be equal to the ratio between the stance (longer segment) and gait cycle duration (i.e., stride time, whole segment)[2]:

$$\phi = \frac{Stance\ time}{Swing\ time} = \frac{\text{Stride time}}{Stance\ time}$$

The value of ϕ close to ~1.618 indicates a relatively harmonic gait, while other values of ϕ ~1.618 indicate an atypical gait harmony. The ratio was calculated separately for the left and right / MAS and LAS as it could also indicate side differences.

Supplementary Note 2 - Stepping Phase Coordination Index (PCI)

We estimated the stepping phase coordination of gait by quantifying the phase relationship between the step durations of the left/MAS and right/LAS sides[8]. This estimation is based on determining the phase ([°], φ_{pci}) and computing the phase coordination index (PCI). To determine the phase, we use the time between the start of a gait cycle and the time point at which the other leg's heel strike occurs. Normalizing the step time with respect to the stride time and scaling by 360° defines the phase of the *ith* stride via the formula:

$$\varphi_{pci} = 360\ x\ \frac{(t_{si} - t_{Li})}{(t_{L\,(i+1)} - t_{Li})}$$

which t_{si} and t_{Li} refer to the time of the *i*th heel strike of short and long swing times (see more details in Supplementary Figure 1). The φ_{pci} refers to a relative cycle timing in which the pre-factor 360 in the formula is used to transform the variable into degrees[8]

After determining the phase, PCI can be computed. PCI incorporates the sum of two percentages values ((A) percentage of the difference between the step and 180° ($P\varphi_{pci}\_ABS$, accuracy) and (B) coefficient of variation of the mean of φ_{pci} (${\varphi_{pci}}_{CV}$, consistency)):

(A) Accuracy: $P\varphi_{pci}\_ABS = 100\,x\left(\frac{\varphi_{pci}\_ABS}{180}\right)$

$$P\varphi_{pci}\_ABS = \overline{|\varphi_{pci} - 180°|}$$

(B) Consistency: $\varphi_{pci}\_CV = \left(\frac{\delta\varphi_{pci}}{\bar{x}\varphi_{pci}}\right)x100$

Where δ and $\bar{x}$ are the standard deviation and the mean of φ_{pci}, respectively.

PCI consists of the sum of $P\varphi_{pci}\_ABS$ and φ_{pci}_CV, reflecting both consistency and accuracy of the left–right stepping timing relationship. Lower PCI values reflect a more consistent and accurate phase generation. Supplementary Figure 2 shows φ_{pci} for a PD-OFF, PD-ON and OA groups.

Supplementary Note 3 - Statistics

We compared cortico-cortical, cortico-muscular, and intermuscular coherences, stride outcomes (stride length, swing phase, stance phase, stride duration, speed), phase coordination index (PCI), and Golden Ratio. We conducted two ANOVAs, firstly, to verify the effects of Medication (PD-OFF vs. PD-ON) and Side (MAS vs. LAS), and, secondly, the effects of Group (PwPD vs. CG) and Side.

Correlation

In general, ANOVA only indicated main effects of Medication. Thus, we selected to conduct correlation analysis considering the Delta ($\Delta = ON - OFF$) for the correlation and gait outcomes in which ANOVA revealed main effects of Medication. The correlation model adopted for the effect of the Medication is fully described in the "*Statistical analysis"* in the body of the manuscript. We however also planned to conduct analysis in case ANOVA revealed relevant significant Side differences, which was not the case in our study.

If relevant Side differences had been observed, we would have correlated Side differences for coherences with gait outcomes. For this purpose, we would have calculated the Asymmetry Index (AI) [formula below] to verify if the AI of stride outcomes and the Golden Ratio would be associated with the AI of coherences. AI would be computed following the formula[9]:

$$AI = 100 * \left|\left(\frac{LAS\ or\ Right - MAS\ or\ Left}{LAS\ or\ Right + \ MAS\ or\ Left}\right)\right|$$

where MAS and LAS represent the more affected and less affected sides, respectively. The more and less affected sides are computed based on the sum of the items 3.3, 3.4, 3.5, 3.6, 3.7, 3.8, 3.15, 3.16, and 3.17) of MDS-UPDRS.

Supplementary Results

Supplementary Table 1 shows the participants' characteristics and functional mobility (i.e., Timed Up and Go [TUG]). Except for age (T_{21}=2.15; p=0.04; d=0.99) and TUG (T_{21}=2.97; p<0.01), PD and OA groups were similar for demographic characteristics. Medication significantly improved functional mobility (PD-ON vs. PD-OFF performed the TUG faster, d=-1.08). Between groups, PD-OFF vs. OA performed TUG ~9 s more slowly (d=-1.3), and PD-ON did not differ from OA's TUG performance. Specifically considering clinical measures, the dopamine intake decreased motor symptoms in the score for the MDS-UPDRS-III (T_{21}=4.03; p<0.01; d=0.55). Note that this result was also reported in the manuscript.

Supplementary Table 2 depicts ANOVA/T-Tests outcomes (F/T, p, partial eta square [$\boldsymbol{\eta_P^2}$]) considering main effect or interaction of Medication (PD-ON vs. PD-OFF), Group (PD-ON or PD-OFF vs. OA), Side (MAS vs. LAS)

Results that did not indicate statistically significant differences are reported here (p>0.05). ANOVAs did not indicate Medication, Side main effect, or interactions between those factors, nor indicate main effect of Group, Side, or interactions between PD-OFF and PD-ON vs. OA for the swing, stance, and duration (p>0.05). Thus, PD-OFF, PD-ON, and OA were similar in swing, stance, and step durations (*Supplementary Figure 3*).

ANOVAs also did not indicate Medication, Side, or Group main effects, nor interactions for the Golden ratio (p>0.05). Unexpectedly, Medication state (PD-OFF vs. PD-ON) and Group (people with Parkinson’s Disease vs. Older Adults) did not interfere with the proportion between stance/swing phases and between gait cycle/stance phases (relatively gait harmonic – values close to Golder Ratio = 1.618, *Supplementary Figure 4*).

Regarding intermuscular coherence, the significant Medication, Side, or Group differences for vastus lateralis (VL)-tibialis anterior (TA) and VL-biceps femoris (BF) coherences for the stance and BF-gastrocnemius lateralis (GL) and VL-TA coherences for the swing are detailed in the main body of the manuscript. However, we did not observe any main effect (Medication, Side, or Group), nor interaction for BF-GL and TA-GL intermuscular coherence

for the stance (p>0.05, Supplementary Figure 5a and 5b) and for VL-BF and TA-GL for the swing (p>0.05, *Supplementary Figure 5c and 5d*).

Supplementary Figures 6-10 depict the distribution of coherence over frequency. Such figures allow the visualization of specific frequencies in which the coherence can differ across groups or sides. Supplementary Figure 6 illustrates cortico-cortical (C3-C4) coherence during the stance and swing phases. Supplementary Figures 7 and 8 represent cortico-muscular coherences over time during the stance and swing phases of walking, respectively. Figures 9 and 10 represent intermuscular coherences over time during stance and swing phases of walking, respectively.

Supplementary Tables

Supplementary Table 1. Participants' characteristics, functional mobility, and global cognitive measures in people with PD and OA.

	**PD**		**OA**
Sex (f/m)	6/8		6/3
Age (years)	65.64 ± 10.42#		73.56 ± 4.39
Height (m)	1.72 ± 0.09		1.66 ± 0.06
Weigh (kg)	72.96 ± 12.6		68.72 ± 5.89
BMI (kg/m²)	24.69 ± 3.58		25.08 ± 2.19
LEDD (mg)	801.83 ± 337.47		N/A
N-FOGQ (score)	16.21 ± 8.51		N/A
	**PD-OFF**	**PD-ON**	**OA**
MoCA (score)	25.29 ± 3.43	24.79 ± 2.94	25.33 ± 2.45
TUG (s)	17.94 ± 7.82#*	11.45 ± 3.23	9.91 ± 2.27
MDS-UPDRS-III (score)	33.21 ± 17.8*	24.36 ± 14.29	0 ± 0

#Group difference: PD ≠ OA (p<0.05); *Medication differences: PD-OFF ≠ PD-ON (p<0.01). The characteristics analyzed were the Montreal Cognitive Assessment (MoCA); Body Mass Index (BMI); Levodopa equivalent daily dose (LEDD); Timed Up and Go (TUG); Modified Unified Parkinson's Disease Rating Scale-Motor Section (MDS-UPDRS-III); and New Freezing of Gait Questionnaire (N-FOGQ). N/A indicates that the characteristic was ‘not assessed’.

Supplementary Table 2. ANOVA outcomes for Medication (PD-ON vs. PD-OFF), Group (PD-ON or PD-OFF vs. OA), Side (MAS vs. LAS) effects, and interactions.

**Outcomes**	**Medication**			**Group**				**Side**			**Side *Medication**			**Side*Group**			
	**T/F**	**p**	η_P^2	**vs. OA**	**T/F**	**p**	η_P^2	**T/F**	**P**	η_P^2	**T/F**	**p**	η_P^2	**vs. OA**	**T/F**	**p**	η_P^2
Gait measures																	
Stride Length	13.15	<0.01	0.5	OFF	14.81	<0.01	0.41	-	-	-	-	-	-	-	-	-	-
Speed	10.4	<0.01	0.44	OFF	10.1	<0.01	0.33	-	-	-	-	-	-	-	-	-	-
PCI	5.76	0.03	0.31	ON<br>OFF	4.73<br>12.83	<0.05	0.92<br>1.78	-	-	-	-	-	-	-	-	-	-
Cortico-cortical coherence																	
C3-C4 Gamma - Stance	-	-	-	-	-	-	-	4.39	0.49	0.17	-	-	-	-	-	-	-
C3-C4 Gamma - Swing	-	-	-	-	-	-	-	-	-	-	9.39	<0.01	0.42	-	-	-	-
Cortico-muscular coherence																	
Ct-VL Alpha - Stance	7.05	0.02	0.35	-	-	-	-	-	-	-	-	-	-	-	-	-	-
Ct-VL Beta - Stance	-	-	-	-	-	-	-	-	-	-	-	-	-	OFF	5.58	0.03	0.21
Ct-VL Gamma - Stance	10.22	<0.01	0.44	-	-	-	-	4.51	<0.05	0.18	-	-	-	ON<br>OFF	9.04<br>9.79	<0.01	0.3<br>0.32
Ct-BF Beta - Stance	-	-	-	-	-	-	-	5.02	0.043	0.28	-	-	-	-	-	-	-
Ct-TA Alpha -Stance	13.19	<0.01	0.5	-	-	-	-	-	-	-	-	-	-	-	-	-	-
Ct-TA Gamma - Stance	5.26	0.04	0.29	-	-	-	-	-	-	-	-	-	-	-	-	-	-
Ct-GL Alpha - Stance	14.84	<0.01	0.53	-	-	-	-	-	-	-	-	-	-	-	-	-	-
Ct-VL Alpha - Swing	13.21	<0.01	0.5	ON	4.36	<0.05	0.17				-	-	-	-	-	-	-
Ct-BF Alpha - Swing	24.46	<0.01	0.65	ON	7.57	0.01	0.27	-	-	-	-	-	-	-	-	-	-
Ct-TA Beta - Swing	5.62	<0.01	0.3											-	-	-	-
Ct-GL Alpha - Swing	12.08	<0.01	0.48	-	-	-	-	-	-	-	-	-	-	-	-	-	-
Ct-GL Beta - Swing	5.16	0.04	0.28	-	-	-	-	-	-	-	-	-	-	ON<br>OFF	4.47<br>7.36	<0.05	0.18<br>0.26
Ct-GL Gamma - Swing	15.99	0.02	0.55	-	-	-	-	-	-	-	-	-	-	-	-	-	-
Intermuscular coherence																	
VL-BF Gamma - Stance	-	-	-	-	-	-	-	-	-	-	-	-	-	ON	8.03	0.01	0.28
VL-TA Alpha - Stance	5.98	0.03	0.32	-	-	-	-	-	-	-	-	-	-	OFF	4.72	0.04	0.18
VL-TA Alpha - Swing	-	-	-	-	-	-	-	-	-	-	-	-	-	ON	4.37	<0.05	0.17
BF-GL Alpha - Swing				OFF	8.64	<0.01	0.29	-	-	-	-	-	-				
BF-GL Beta - Swing	6.53	0.024	0.33	-	-	-	-	-	-	-	-	-	-	-	-	-	-
BF-GL Gamma - Swing	4.51	0.053	0.26	-	-	-	-	-	-	-	-	-	-	-	-	-	-

T/F values represent the values for T-Tests and ANOVA comparison; η_P^2: partial eta square

Supplementary Figures

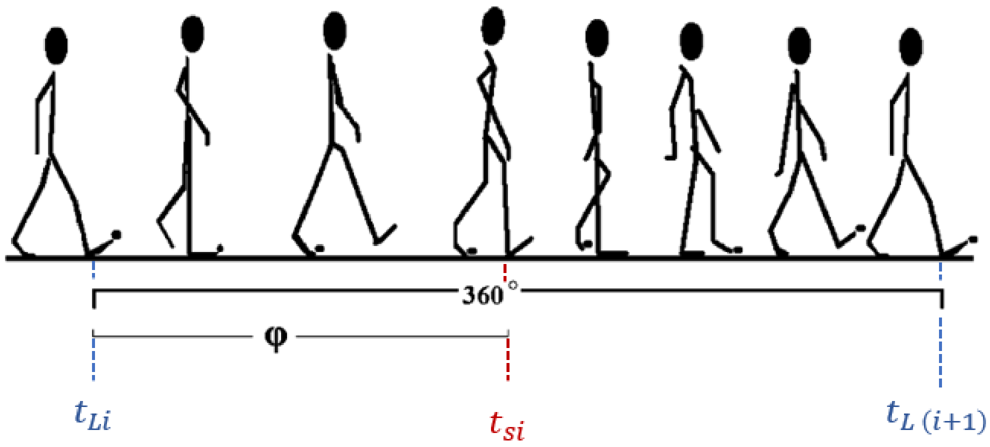

Supplementary Figure 1. Schematic illustration of how the stepping phases generation (φ_{pci}) is defined. In this example, the right leg (R) is the leg with the long swing times on average, and, therefore, is the leg used as the reference. Thus, this figure depicts how short (t_s) and long swing times (t_L) of a specific gait cycle (ith) were denoted for computing the formula $\varphi_{pci} = 360\ x\ (t_{si} - t_{Li})/(t_{L\ (i+1)} - t_{Li})$. The figure was adapted from Plotnik, Giladi and Hausdorff[8]

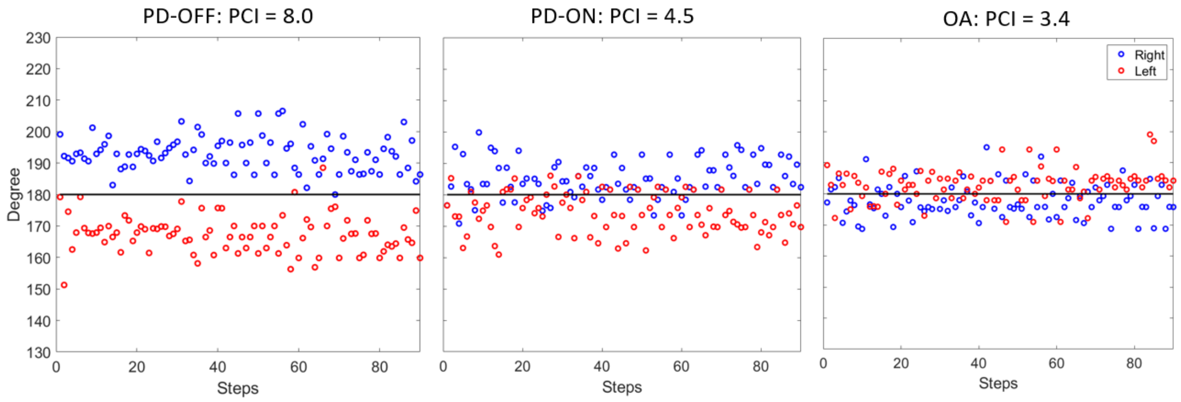

Supplementary Figure 2. Stepping phase values are plotted for a participant with PD at OFF medication state (PD-OFF, left), a participant with PD at ON medication state (PD-ON middle) and a healthy Older adult (OA, right). In this example, while for the OA, stepping phases are scattered close to the 180° line, for PD-ON and PD-OFF, stepping phases are relatively more distant from 180° than the OA.

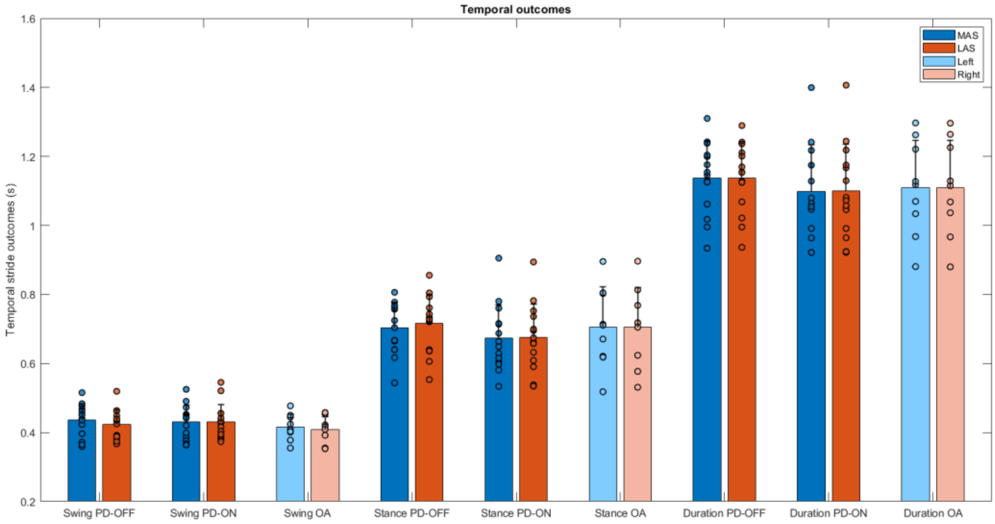

Supplementary Figure 3. Means (bars), standard deviations (error bars), and individual values (circles) for temporal stride outcomes (swing, stance and duration) for people with Parkinson's Disease at OFF (PD-OFF) and ON medication states (PD-ON) and healthy Older adults (OA). Blue and red bars and dots mean the More Affected Side (MAS) and Less Affected Side (LAS), respectively. Please note that for the OA group, the light blue and red colors represent the Left and Right sides, respectively. Please note that for the OA group, the light blue and red colors represent the Left and Right sides, respectively.

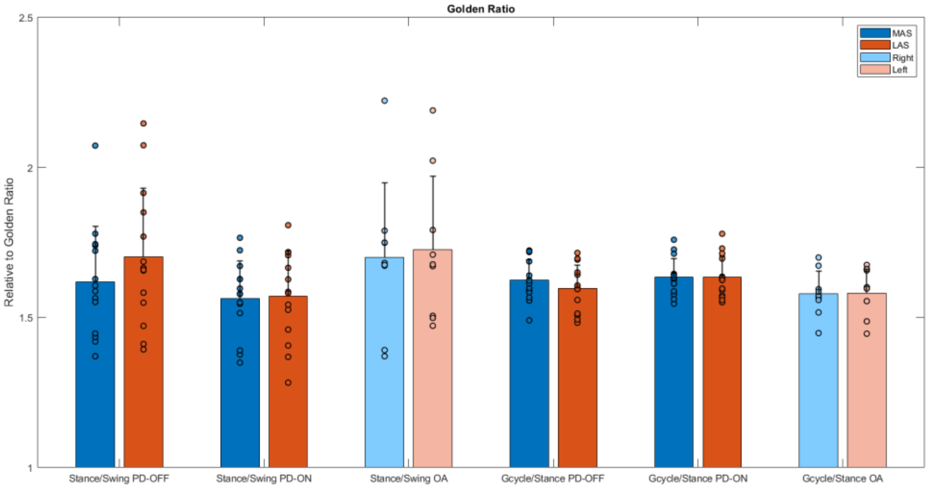

Supplementary Figure 4. Means (bars), standard deviations (error bars), and individual values (dots) for the Golden Ratio (proportions between stance/swing phases and gait cycle (Gcycle)/stance phases) for people with Parkinson's Disease at OFF (PD-OFF) and ON medication state (PD-ON) and healthy older adults (OA). Blue and red bars and dots mean

more affected (MAS) and less affected sides (LAS), respectively. Please note that for the OA group, the light blue and red colors represent the Left and Right sides, respectively.

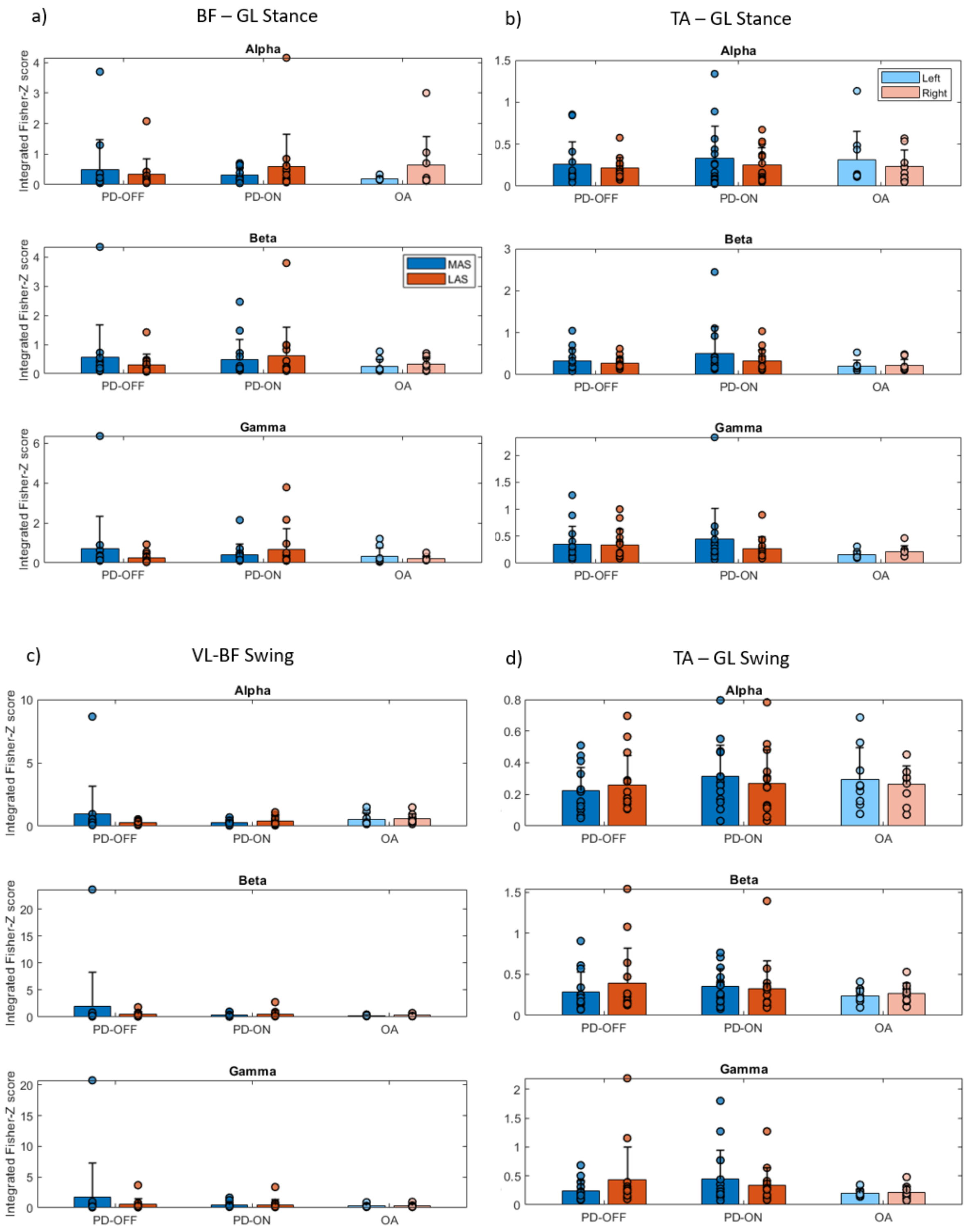

Supplementary Figure 5. Means (bars), standard deviations (error bars), and individual values (dots) for cumulative intermuscular coherence between (a) biceps femoris (BF)-gastrocnemius lateralis (GL) for stance, (b) tibialis anterior (TA)-GL for stance, (c) vastus

lateralis (VA)-BF for swing, and (d) TA-GL for swing. Blue and red bars and dots mean the More Affected Side (MAS) and Less Affected Side (LAS), respectively. Please note that for the OA group, the light blue and red colors represent the Left and Right sides, respectively.

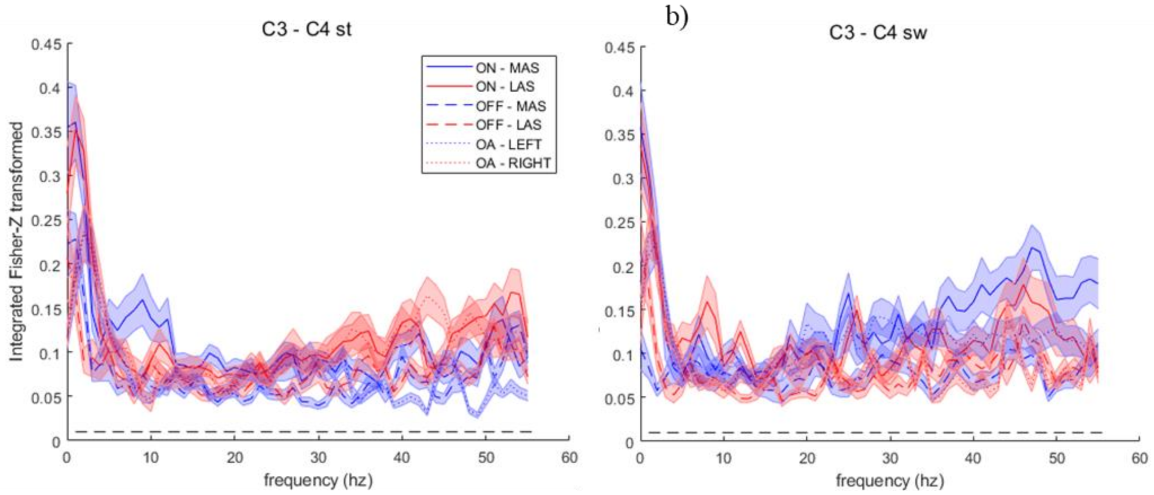

Supplementary Figure 6. Cortico-cortical (C3-C4) coherence across frequency during (a) stance and (b) swing phases. Continuous, dashed, and dotted lines represent PD at ON, OFF, and OA, respectively. Shaded areas represent standard error. Blue and Red colors represent the Most Affected Aide (MAS) and Less Affected Side (LAS) for PD, and the Left and Right sides for OA, respectively.

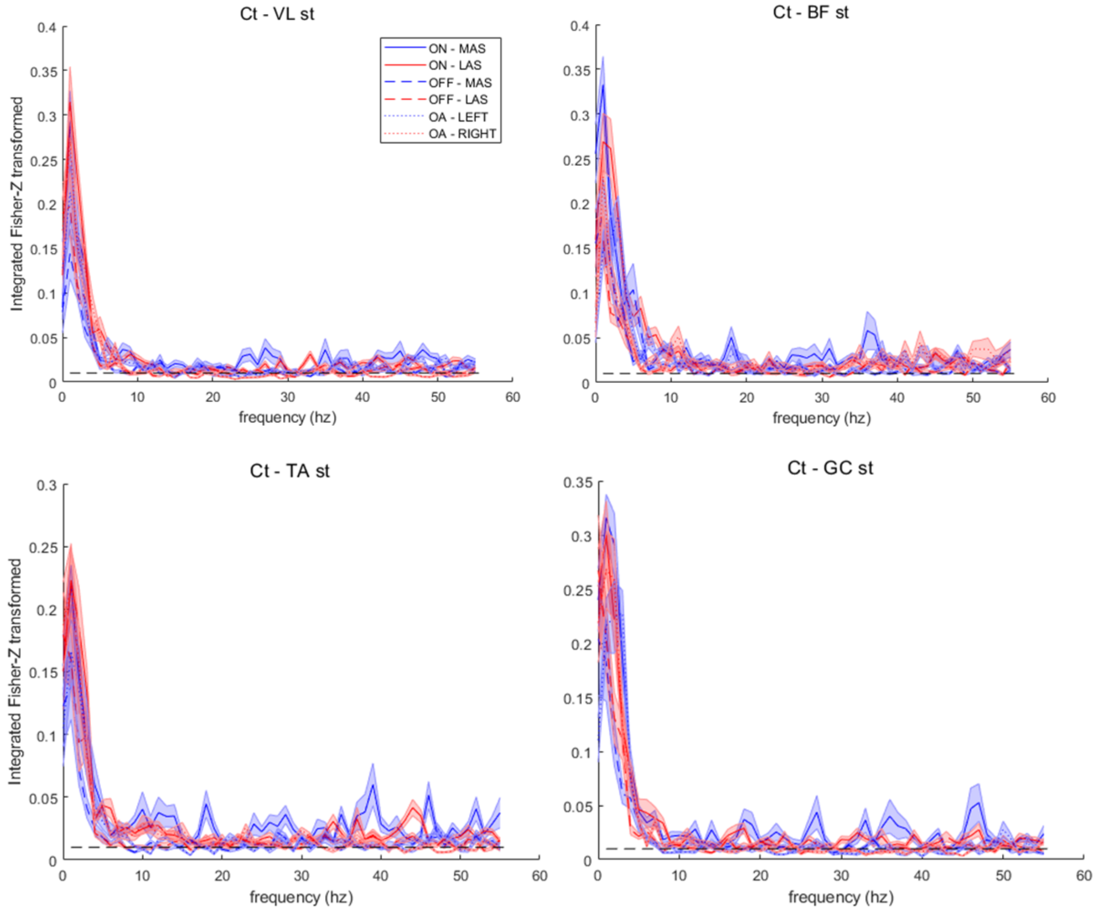

Supplementary Figure 7. Cortico-muscular coherences across frequency during the stance phase (st). Continuous, dashed, and dotted lines represent PD at ON, OFF, and OA, respectively. Shaded areas represent standard error. Blue and red colors represent the Most Affected Aide (MAS) and Less Affected Side (LAS) for PD, and the Left and Right sides for OA, respectively. Ct: cortical, VL: vastus lateralis, BF: biceps femoris, TA: tibial anterior, GC: gastrocnemius lateralis.

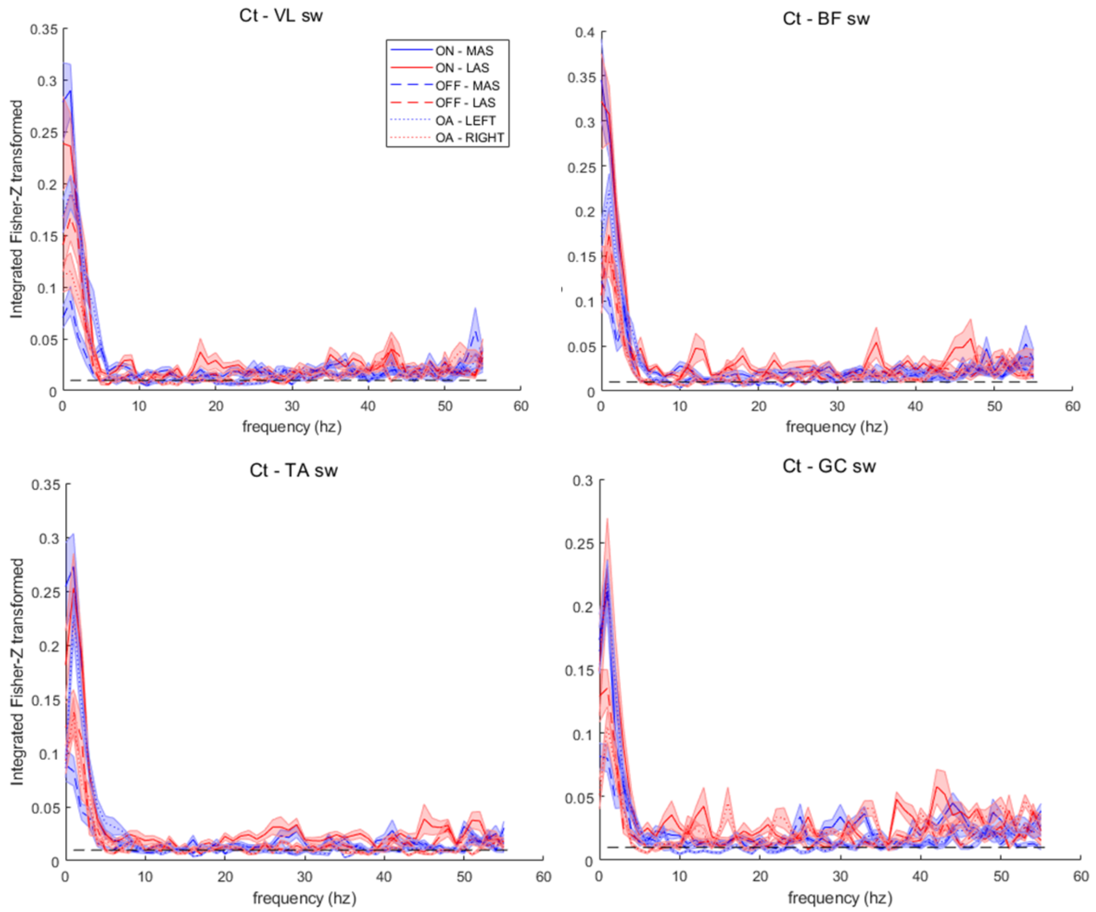

Supplementary Figure 8. Cortico-muscular coherences across frequency during the swing phase (sw). Continuous, dashed, and dotted lines represent PD at ON, OFF, and OA, respectively. Shaded areas represent standard error. Blue and red colors represent the Most Affected Aide (MAS) and Less Affected Side (LAS) for PD, and the Left and Right sides for OA, respectively. Ct: Cortical, VL: vastus lateralis, BF: biceps femoris, TA: tibial anterior, GC: gastrocnemius lateralis.

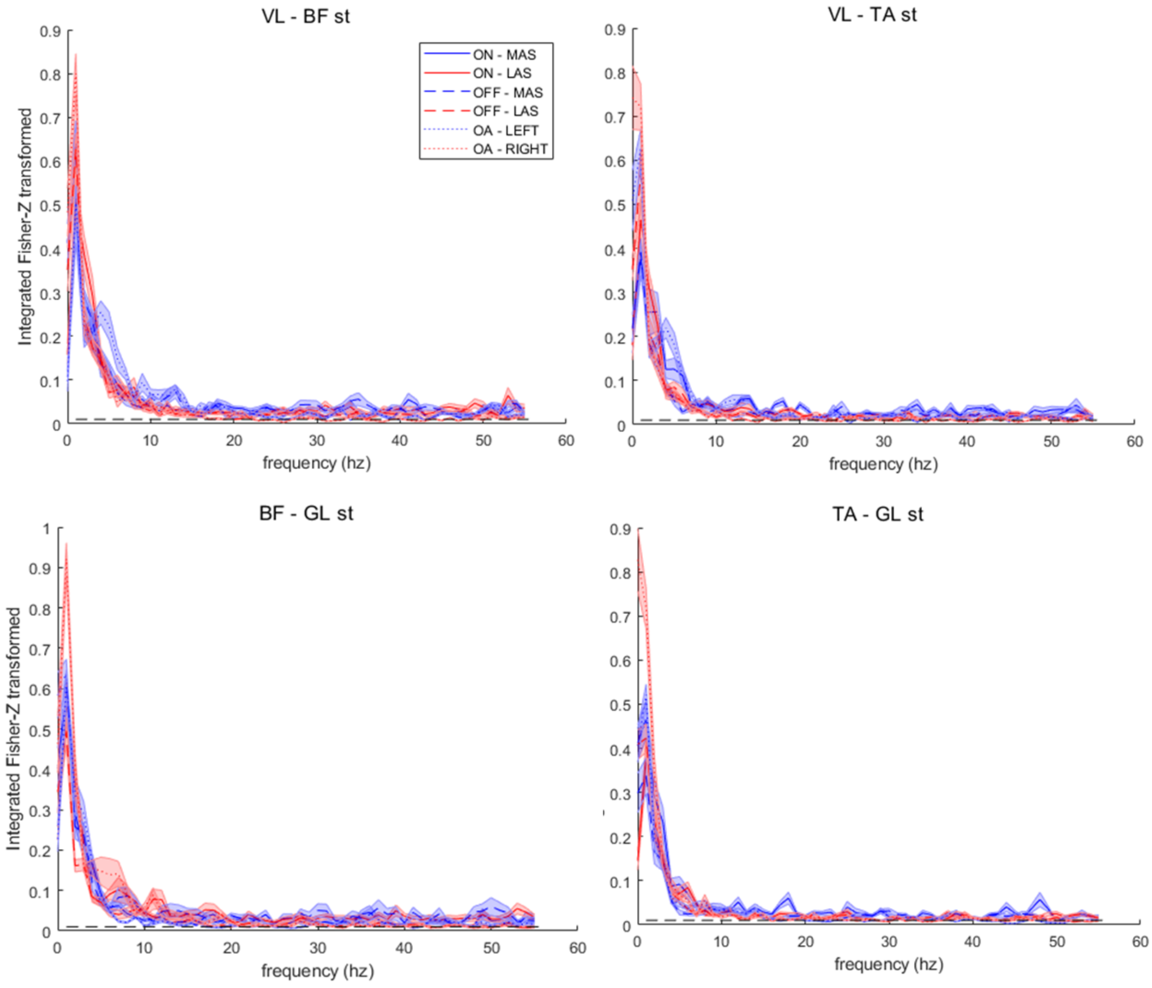

Supplementary Figure 9. Intermuscular coherence across frequency during the stance phase (st). Continuous, dashed, and dotted lines represent PD at ON, OFF, and OA, respectively. Shaded areas represent standard error. Blue and red represent the Most Affected Aide (MAS) and Less Affected Side (LAS) for PD, and the Left and Right sides for OA, respectively. VL: Vastus Lateralis, BF: biceps femoris, TA: tibial anterior, GL: gastrocnemius lateralis.

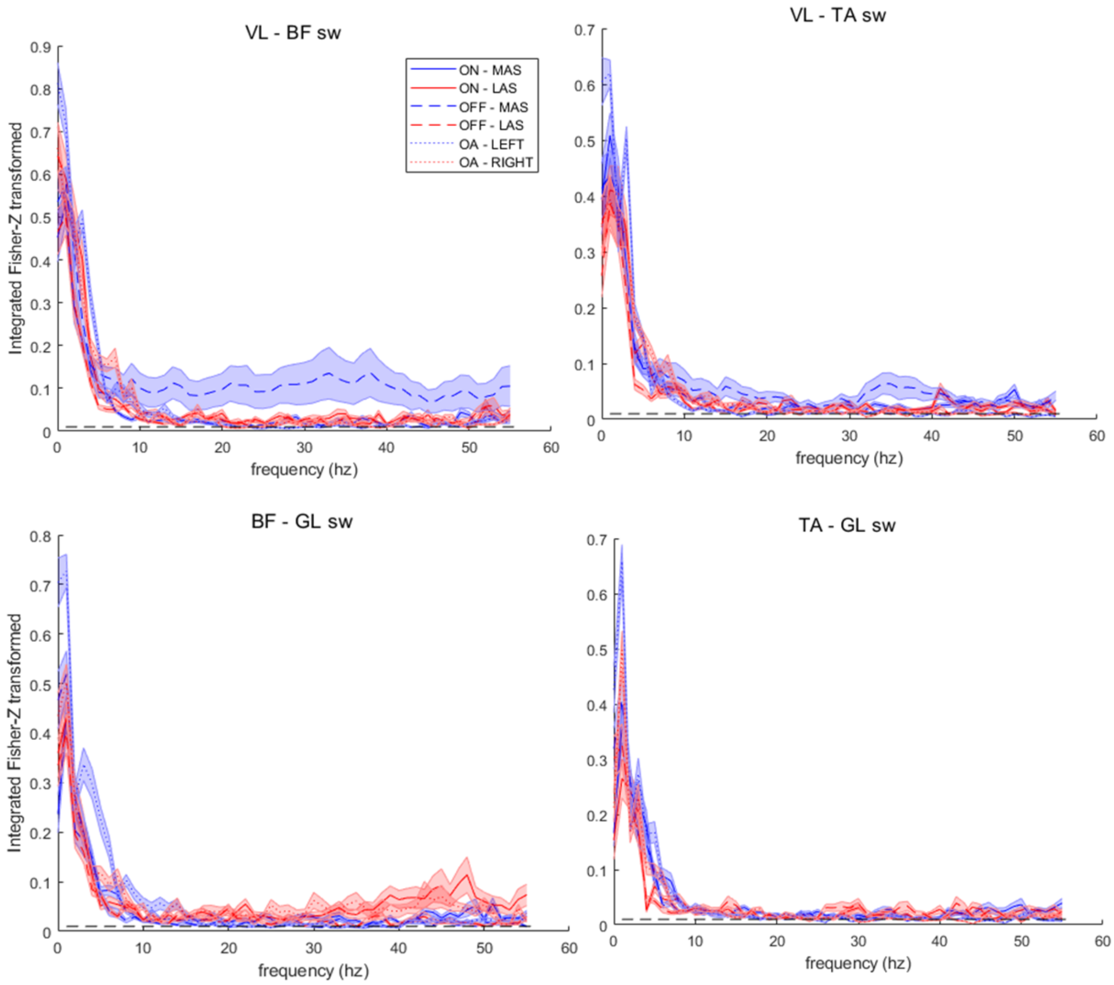

Supplementary Figure 10. Intermuscular coherence across frequency during the swing phase (sw). Continuous, dashed, and dotted lines represent PD at ON, OFF, and OA, respectively. Shaded areas represent standard error. Blue and red represent the Most Affected Aide (MAS) and Less Affected Side (LAS) for PD, and the Left and Right sides for OA, respectively. VL: vastus lateralis, BF: biceps femoris, TA: tibial anterior, GL: gastrocnemius lateralis.

Supplementary References

1. Iosa, M. *et al.* Loss of fractal gait harmony in Parkinson's Disease. *Clin. Neurophysiol.* **127**, 1540–1546 (2016).
2. Iosa, M. *et al.* The golden ratio of gait harmony: repetitive proportions of repetitive gait phases. *Biomed Res. Int.* **2013**, (2013).

3. Serrao, M. *et al.* Harmony as a convergence attractor that minimizes the energy expenditure and variability in physiological gait and the loss of harmony in cerebellar ataxia. *Clin. Biomech.* **48**, 15–23 (2017).

4. Iosa, M., Marro, T., Paolucci, S. & Morelli, D. Stability and harmony of gait in children with cerebral palsy. *Res. Dev. Disabil.* **33**, 129–135 (2012).

5. Grattan-Guinness, I. *Companion Encyclopedia of the History and Philosophy of the Mathematical Sciences*. *Companion Encyclopedia of the History and Philosophy of the Mathematical Sciences* (Routledge, 2002). doi:10.4324/9780203014585/COMPANION-ENCYCLOPEDIA-HISTORY-PHILOSOPHY-MATHEMATICAL-SCIENCES-IVOR-GRATTAN-GUINNESS.

6. Okabe, T. Physical phenomenology of phyllotaxis. *J. Theor. Biol.* **280**, 63–75 (2011).

7. Coldea, R. *et al.* Quantum criticality in an Ising chain: experimental evidence for emergent E8 symmetry. *Science* **327**, 177–180 (2010).

8. Plotnik, M., Giladi, N. & Hausdorff, J. M. A new measure for quantifying the bilateral coordination of human gait: effects of aging and Parkinson's disease. *Exp. Brain Res. 2007 1814* **181**, 561–570 (2007).

9. Orcioli-Silva, D. *et al.* Double obstacles increase gait asymmetry during obstacle crossing in people with Parkinson's disease and healthy older adults: A pilot study. *Sci. Rep.* **10**, 1–8 (2020).
