# Supplementary material for: Dopamine improves defective cortical and muscular connectivity during bilateral control of gait in Parkinson’s disease
Source: Commun Biol. 2024 Apr 24;7:495. doi: 10.1038/s42003-024-06195-5 (PMC11043351; doi:10.1038/s42003-024-06195-5)
Supplement: Supplementary file 3 — Description of Additional Supplementary Files [file 42003_2024_6195_MOESM3_ESM.pdf]

Description of Additional Supplementary Files

File Name: Supplementary Data

Description: The source data used to generate plots and statistical analysis.
