# Supplementary material for: Dopamine improves defective cortical and muscular connectivity during bilateral control of gait in Parkinson’s disease
Source: Commun Biol. 2024 Apr 24;7:495. doi: 10.1038/s42003-024-06195-5 (PMC11043351; doi:10.1038/s42003-024-06195-5)
Supplement: Supplementary file 5 — Reporting Summary [file 42003_2024_6195_MOESM5_ESM.pdf]

nature portfolio

Corresponding author(s): Paulo Cezar Rocha dos Santos

Last updated by author(s): Apr 2, 2024

Reporting Summary

Nature Portfolio wishes to improve the reproducibility of the work that we publish. This form provides structure for consistency and transparency in reporting. For further information on Nature Portfolio policies, see our Editorial Policies and the Editorial Policy Checklist.

Statistics

For all statistical analyses, confirm that the following items are present in the figure legend, table legend, main text, or Methods section.

n/a	Confirmed	
☐	☒	The exact sample size (*n*) for each experimental group/condition, given as a discrete number and unit of measurement
☒	☐	A statement on whether measurements were taken from distinct samples or whether the same sample was measured repeatedly
☐	☒	The statistical test(s) used AND whether they are one- or two-sided<br>*Only common tests should be described solely by name; describe more complex techniques in the Methods section.*
☒	☐	A description of all covariates tested
☐	☒	A description of any assumptions or corrections, such as tests of normality and adjustment for multiple comparisons
☐	☒	A full description of the statistical parameters including central tendency (e.g. means) or other basic estimates (e.g. regression coefficient) AND variation (e.g. standard deviation) or associated estimates of uncertainty (e.g. confidence intervals)
☐	☒	For null hypothesis testing, the test statistic (e.g. *F*, *t*, *r*) with confidence intervals, effect sizes, degrees of freedom and *P* value noted<br>*Give P values as exact values whenever suitable.*
☒	☐	For Bayesian analysis, information on the choice of priors and Markov chain Monte Carlo settings
☒	☐	For hierarchical and complex designs, identification of the appropriate level for tests and full reporting of outcomes
☐	☒	Estimates of effect sizes (e.g. Cohen's *d*, Pearson's *r*), indicating how they were calculated

Our web collection on statistics for biologists contains articles on many of the points above.

Software and code

Policy information about availability of computer code

Data collection

For the data collection, the following devices and softwares were used:
- 6 OPAL sensors (OPAL, APDM WEARABLE TECHNOLOGIES INC., Portland, Oregon, USA.
- 8 channels Electromyography (sEMG) recording system (Eego referential amplifier, eemagine Medical Imaging Solutions GmbH, Berlin, Germany) - ANT Neuro (ANT Neuro, Hengelo, the Netherlands).
- 32-channel portable EEG cap (EEGO Sports™, eemagine Medical Imaging Solutions GmbH, Berlin, Germany) - ANT Neuro (ANT Neuro, Hengelo, the Netherlands).

We also used the following questionnaires/scales
- Montreal Cognitive Assessment (MoCA, Hebrew validated version);
- Modified Unified Parkinson's Disease Rating Scale-Motor Section (MDS-UPDRS)
- Timed Up and Go
- New Freezing of Gait Questionnaire (N-FOGQ)

Data analysis

For the data analysis, we used:
- MATLAB (v.22a, The MathWorks Inc., Natick, MA);
- SPSS for Windows (Version 25, IBM, Armonk, NY, USA);
- R-Studio (Version 2022.02., Boston, USA).

For manuscripts utilizing custom algorithms or software that are central to the research but not yet described in published literature, software must be made available to editors and reviewers. We strongly encourage code deposition in a community repository (e.g. GitHub). See the Nature Portfolio guidelines for submitting code & software for further information.

Data

Policy information about availability of data

All manuscripts must include a data availability statement. This statement should provide the following information, where applicable:

- Accession codes, unique identifiers, or web links for publicly available datasets
- A description of any restrictions on data availability
- For clinical datasets or third party data, please ensure that the statement adheres to our policy

The data generated or analyzed during this study are included in this published article and its Supplementary Information Files. Additional information generated during the current study is available from the corresponding author upon reasonable request from the corresponding author.

Research involving human participants, their data, or biological material

Policy information about studies with human participants or human data. See also policy information about sex, gender (identity/presentation), and sexual orientation and race, ethnicity and racism.

Reporting on sex and gender

Individuals from both sexes (male and female) composed our sample. The information was included in the table that reported the characteristics of the participants.

Reporting on race, ethnicity, or other socially relevant groupings

Our study included people with Parkinson's disease and healthy older adults, independent of race and ethnicity

Population characteristics

We reported all relevant characteristics in table 1 in the body of the manuscript.

Recruitment

Individuals with PD were recruited from the Movement Disorders and the Neurological Rehabilitation Institutes at Sheba Medical Center. Healthy controls were invited by word of mouth.

Ethics oversight

The experimental protocol was approved by the Sheba Medical Center Institutional Review Board

Note that full information on the approval of the study protocol must also be provided in the manuscript.

Field-specific reporting

Please select the one below that is the best fit for your research. If you are not sure, read the appropriate sections before making your selection.

☒ Life sciences ☐ Behavioural & social sciences ☐ Ecological, evolutionary & environmental sciences

For a reference copy of the document with all sections, see nature.com/documents/nr-reporting-summary-flat.pdf

Life sciences study design

All studies must disclose on these points even when the disclosure is negative.

Sample size

Initially, data sets from 28 people with PD and 10 healthy OA had the potential to be included. After applying exclusion criteria, the final sample size was comprised of 14 people with PD and 9 OA. The sample size was based on the availability of participants collected by the center that reached the inclusion criteria.

Data exclusions

Inclusion criteria were diagnosis of idiopathic PD, 50-90 years of age, being under levodopa treatment, able to walk unassisted and without pain for at least 100m, and participation in the protocol during ON and OFF conditions (in two separate visits, 2-3 weeks apart). During data collection, the exclusion criteria were as follows: surgery within the last 6 months or brain surgery at any point in the past; history of stroke: severe peripheral neuropathy, with symptomatic lumbar spinal stenosis; and serious co-morbidities that affect gait and capacity to perform the protocol. Since the present study relies on electroencephalography (EEG) and surface electromyography (sEMG) data, additional exclusion criteria related to the data analysis were adopted, such as rejection of EEG-channels C3 or C4 during signal preprocessing, higher cortico-cortical coherence in all bands (indicative of cross-talk), and lastly, high coherence that was not consistent with near-zero lag synchronization (suggesting cross-talk)

Replication

Not applicable

Randomization	Not applicable
Blinding	Not applicable

Reporting for specific materials, systems and methods

We require information from authors about some types of materials, experimental systems and methods used in many studies. Here, indicate whether each material, system or method listed is relevant to your study. If you are not sure if a list item applies to your research, read the appropriate section before selecting a response.

Materials & experimental systems

n/a	Involved in the study
☒	☐ Antibodies
☒	☐ Eukaryotic cell lines
☒	☐ Palaeontology and archaeology
☒	☐ Animals and other organisms
☐	☒ Clinical data
☒	☐ Dual use research of concern
☒	☐ Plants

Methods

n/a	Involved in the study
☒	☐ ChIP-seq
☒	☐ Flow cytometry
☒	☐ MRI-based neuroimaging

Clinical data

Policy information about clinical studies

All manuscripts should comply with the ICMJE guidelines for publication of clinical research and a completed CONSORT checklist must be included with all submissions.

Clinical trial registration	Not applicable
Study protocol	Not applicable
Data collection	This is a comparative study in which we verified the effects of dopamine medication and side (right and left) on cortico-muscular couplings (inferred via cortico-cortical, cortico-muscular, and intermuscular coherence) and on gait metrics in people with Parkinson's disease under ON (PD-ON) and OFF medication state (PD-OFF) and older adults (OA). We assessed the cortical activity and muscle activity by registering electroencephalography and electromyography and the gait metrics using inertial sensors.
Outcomes	Primary outcome: Cortico-muscular coherence (as we expected that medication would improve the cortical control of muscle during gait);Secondary outcomes: Cortico-cortical coherence, intermuscular coherence; Gait metrics (stride length, width, duration, speed, phase coordination index, golden ratio)

Plants

Seed stocks	Not applicable
Novel plant genotypes	Not applicable
Authentication	Not applicable
